# Maternal folate genes and aberrant DNA hypermethylation in pediatric acute lymphoblastic leukemia

**DOI:** 10.1371/journal.pone.0197408

**Published:** 2018-05-15

**Authors:** Jeremy M. Schraw, Teresa T. Yiu, Philip J. Lupo, Spiridon Tsavachidis, Rachel Rau, Melissa L. Bondy, Karen R. Rabin, Lanlan Shen, Michael E. Scheurer

**Affiliations:** 1 Dan L. Duncan Cancer Center, Baylor College of Medicine, Houston, Texas, United States of America; 2 Department of Pediatrics, Baylor College of Medicine, Houston, Texas, United States of America; 3 Texas Children’s Cancer and Hematology Centers, Houston, Texas, United States of America; 4 Section of Epidemiology and Population Sciences, Department of Medicine, Baylor College of Medicine, Houston, Texas, United States of America; 5 Center for Cell and Gene Therapy, Baylor College of Medicine, Houston, Texas, United States of America; 6 Stem Cells and Regenerative Medicine Center, Baylor College of Medicine, Houston, Texas, United States of America; 7 USDA/ARS Children’s Nutrition Research Center, Baylor College of Medicine, Houston, Texas, United States of America; National Health Research Institutes, TAIWAN

## Abstract

**Background:**

There is evidence that maternal genotypes in folate-related genes are associated with pediatric acute lymphoblastic leukemia (ALL) independent of offspring genotype. We evaluated the relationship between maternal genotypes in methionine synthase (*MTR*) and DNA methylation status in ALL to better characterize the molecular mechanism underlying this association.

**Procedure:**

We obtained bone marrow samples from 51 patients with ALL at diagnosis and from 6 healthy donors. Mothers of patients provided a saliva sample and were genotyped at 11 tagSNPs in *MTR*. DNA methylation was measured in bone marrow mononuclear cells of patients and six healthy marrow donors. We used hierarchical clustering to identify patients with a hypermethylator phenotype based on 281 differentially methylated promoter CpGs. We used logistic regression to estimate the effects of maternal genotype on the likelihood of DNA hypermethylation in ALL and Ingenuity Pathway Analysis to identify networks enriched for differentially methylated genes.

**Results:**

Twenty-two cases (43%) demonstrated promoter hypermethylation, which was more frequent among those with *ETV6-RUNX1* fusion and initial white blood cell count < 50 x 10^9^/L. Maternal rs12759827 was associated with aberrant DNA methylation (odds ratio [OR] 4.67, 95% confidence interval 1.46–16.31); non-significantly elevated ORs were observed for all other SNPs. Aberrantly methylated promoter CpGs aligned to genes with known cancer-related functions.

**Discussion:**

Maternal folate metabolic genotype may be associated with DNA methylation patterns in ALL in their offspring. Therefore, the effect of maternal genotypes on ALL susceptibility may act through aberrant promoter methylation, which may contribute to the *in utero* origins of ALL.

## Introduction

Human tumors are marked by global DNA hypomethylation [1] and gene-specific hypermethylation of tumor suppressors [2]. Studies in pediatric acute lymphoblastic leukemia (ALL) have recently demonstrated that genome-wide DNA methylation profiles are associated with transcriptome changes and clinical outcomes [3]. However, the phenomenon of simultaneous methylation of multiple genes, termed CpG island methylator phenotype (CIMP), which has been best described in colon cancer, has not been systematically evaluated in ALL [4].

ALL can arise as early as infancy, and its incidence peaks between 2 to 4 years of age, suggesting that *in utero* exposures likely modify the risk of ALL in many cases. Evidence from identical twins and neonatal blood spots confirms a prenatal origin of oncogenic abnormalities [5–8]. Moreover, there is some evidence that prenatal folic acid supplementation [9] and genetic variations of folate metabolic genes [10, 11] may reduce the risk of pediatric ALL.

Disrupted maternal folate metabolism may contribute to ALL, possibly by directly affecting the likelihood of preleukemic events *in utero*, or indirectly through cooperating events such as aberrant DNA methylation. We previously showed that maternal 5-methyltetrahydrofolate-homocysteine methyltransferase (*MTR*) genotype was associated with ALL independent of offspring genotype [12]. *MTR* catalyzes the conversion of homocysteine to methionine for synthesis of S-adenosylmethionine (SAM), the methyl donor for DNA methylation. Since DNA methylation is dependent on folate metabolism [13], we evaluated the association between maternal *MTR* genotype and CIMP status among patients with ALL.

## Materials and methods

### Study population

The study population and inclusion criteria have previously been described [11, 12]. Briefly, the current study was performed in a sample of 51 case-mother pairs who had previously been genotyped for variants in *MTR* and for whom cryopreserved bone marrow was available, as well as a convenience sample of six healthy pediatric bone marrow donors (controls) all of whom were seen at Texas Children’s Hospital (Houston, TX, USA) from 2005–2010. Both males and females, and individuals of all race/ethnic groups were eligible. Written informed consent was obtained from parents. We obtained saliva samples from mothers and bone marrow from healthy donors and children with newly diagnosed ALL. Clinical data were abstracted from medical records. Samples were obtained with informed consent under protocols approved by the Baylor College of Medicine Institutional Review Board.

### Genome-wide DNA methylation profiling

DNA was extracted from bone marrow mononuclear cells isolated by Ficoll density centrifugation using the QIAamp DNA Blood Mini Kit (Qiagen, Valencia, CA). After confirmation of the efficiency and accuracy of methylation-specific restriction enzyme digestion, samples were sequenced by methylated CpG island amplification sequencing (MCA-Seq) using an Illumina HiSeq 2000. We randomly selected six genes for validation by pyrosequencing. Data have been deposited in the Gene Expression Omnibus database (GSE99793) [14]. For validation of methylation status in these samples, DNA was bisulfite converted using EpiTect Bisulfite kit (Qiagen, Valencia, CA) and assayed using pyrosequencing (S1 Fig).

We constructed a reference library of human genome hg19 with 10,726 autosomal SmaI/XmaI sites ≤ 2kb in length, within CpG islands (CGIs) and non-repeat regions where methylation was relevant to gene expression and did not differ by sex [15]. A mean of 6,287,008 unique reads per sample were mapped to hg19 and scaled in per million reads. Methylation values for all sites and samples were log-transformed for further analyses.

### Single nucleotide polymorphism (SNP) selection and genotyping methods

We selected 11 tagSNPs in *MTR*, all of which we previously showed to be associated with ALL through the maternal genetic effects [12]. Additionally, there is evidence in the Genotype-Tissue Expression Project (GTEx)[16] that each of these SNPs is associated with MTR expression (p < 5 x 10^−8^) across multiple tissues. As described previously, the SNPs were selected using an r^2^ threshold of 0.80 and the MultiPop-TagSelect Algorithm in the Genome Variation Server. DNA was extracted from maternal saliva samples using the QIAamp DNA Blood Mini Kit. Genotyping was performed using the Sequenom MassARRAY iPLEX platform (Agena Bioscience, San Diego, CA). S1 and S2 Tables list primer sequences used in pyrosequencing and maternal genotyping, respectively.

### Statistical analyses

First, we assessed DNA methylation differences between cases (children with ALL, N = 51) and controls (healthy donors, N = 6) using Student’s *t*-test with Benjamini-Hochberg correction [17]. We identified the top 1,000 differentially methylated autosomal sites residing within CGIs and non-repeat regions with the largest variance among the cases (*sd* >10 and *fdr* <0.1). Secondly, since promoter methylation is often associated with reduced gene expression [18] we subsequently restricted our analyses to promoters. We defined a site as a “promoter” if residing within -1kb to +0.5kb from the transcription start site. We hierarchically clustered the samples on the basis of methylation density at all promoter CpG sites within the list of the top 1,000 differentially methylated sites (N = 281) using Euclidean distance and complete linkage. This method has previously been used to characterize a hypermethylator phenotype in T-lineage ALL [19]. These promoters were subsequently annotated to 208 genes and analysis of pathway, network, and related gene functions was performed using Ingenuity Pathway Analysis (IPA, http://www.ingenuity.com).

Logistic regression was used to calculate odds ratios (OR) and 95% confidence intervals (CI) to evaluate the associations between maternal genotype and promoter methylation after adjustment for ethnicity (non-Hispanic vs. Hispanic). Because of the sample size and the frequency of the minor allele for each SNP, a dominant model of inheritance was used when determining the associations between maternal genotype and CIMP+ phenotype among patients with ALL.

## Results

### DNA methylation

**Fig 1:**
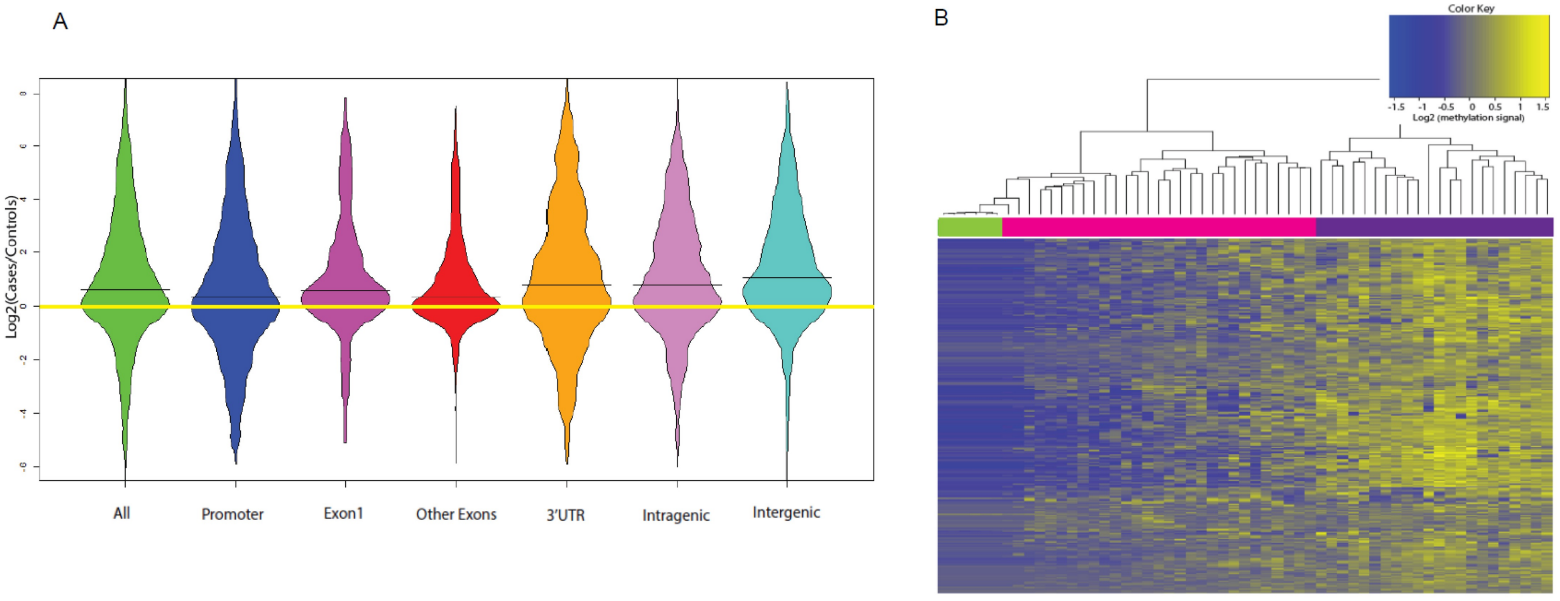
Differential DNA methylation patterns according to case-control status and maternal *MTR* rs12759827 genotype. **(A)** Log ratio of methylation density comparing cases to controls across all genomic locations. Horizontal black lines represent the log ratio of mean methylation density in cases compared to controls. **(B)** Hierarchical clustering demonstrates differential promoter methylation between controls (green) and CIMP- cases (pink) compared to CIMP+ cases (purple).

A mean of 6,287,008 unique reads per sample were mapped to hg19 (summary of sequencing statistics listed in S3 Table). We randomly selected six genes and validated MCA-Seq data with pyrosequencing (S1 Fig). Among the top 1,000 differentially methylated CpG sites, 28.1% resided in promoters, 2.5% in the first exon, 6.4% in other exons, 23.5% in the 3’UTR, 17.9% in intragenic regions and 21.6% in intergenic regions. For all genomic locations, case/control methylation log ratios were > 0 (Fig 1A).

When we performed unsupervised hierarchical clustering using Euclidean distance and complete linkage based on log methylation density the final set of 281 promoter methylation sites, participants segregated into distinct clusters. Cases with low methylation (defined as CIMP-, in pink) clustered separately from cases with relatively higher methylation levels (defined as CIMP+, in purple) as shown by the two distinct branches at the top of dendrogram in Fig 1B. These two groups represent the coarsest clusters chosen by the algorithm. Controls (in green) showed a homogenous pattern of relatively lower methylation density, clustered closely with one another and apart from cases at fine levels of clustering, and with CIMP- cases at coarser levels of clustering.

Table 1 lists demographic and clinical characteristics of patients by CIMP status. Patients in CIMP- and CIMP+ methylation groups were similar with respect to sex, race, ethnicity and clinical outcomes. Patients with CIMP+ had a higher frequency of *ETV6-RUNX1* fusion compared to patients with CIMP- (31.8% and 6.9%, respectively; p = 0.02). Additionally, all cases with initial white blood cell (WBC) count >50 x 10^9^/L were CIMP- (N = 7, P = 0.01).

**Table 1:** Demographic and clinical information of pediatric all patients.

Characteristics	Total *N* = 51	CIMP- *N* = 29	CIMP+ *N* = 22	*P*-value^a^
Age in years, median (IQR)	4 (5.5)	4 (7)	5 (5)	0.36
Male, *N* (%)	26 (51.1%)	15 (51.7%)	11 (50.0%)	0.9
Race-ethnicity				0.38
Hispanic, *N* (%)	32 (62.8%)	19 (65.5%)	13 (59.1%)	
Non-Hispanic White, *N* (%)	16 (31.4%)	7 (24.1%)	9 (40.9%)	
Non-Hispanic Black, *N* (%)	2 (3.9%)	2 (6.9%)	0 (0.0%)	
Other, *N* (%)	1 (2.0%)	1 (3.5%)	0 (0.0%)	
Subtypes				0.62
B-lineage, *N* (%)	47 (92.2%)	26 (89.7%)	21 (95.5%)	
T-lineage, *N* (%)	4 (7.8%)	3 (10.3%)	1 (4.6%)	
Cytogenetics				
Normal/Other^b^	7 (13.7%)	4 (13.8%)	3 (13.6%)	0.99
Hyperdiploid^c^, *N* (%)	22 (43.1%)	13 (44.8%)	9 (40.9%)	1
*ETV6-RUNX1*, *N* (%)	9 (17.6%)	2 (6.9%)	7 (31.8%)	0.02
*CDKN2A* deletion, *N* (%)	11 (21.6%)	7 (24.1%)	4 (18.2%)	0.74
*BCR-ABL1*, *N* (%)	2 (3.9%)	2 (6.9%)	0 (0.0%)	0.5
Initial WBC count >50 x 10^9^/L, *N* (%)	7 (13.7%)	7 (24.1%)	0 (0.0%)	0.01
Relapse	4 (7.8%)	3 (10.3%)	1 (4.6%)	0.62
BMT	4 (7.8%)	2 (6.9%)	2 (9.1%)	1
Death	3 (5.9%)	2 (6.9%)	1 (4.6%)	1

^a^ p-value for difference in median age from Mann-Whitney U test; p-values for differences in proportions from Fisher’s exact test.

^b^ Cytogenetically normal or negative for hyperdiploidy, *ETV6-RUNX1*, *CDKN2A* deletion and *BCR-ABL1*.

^c^ Hyperdiploid defined as > 46 chromosomes by either chromosome analysis, FISH, or both.

Abbreviations: ALL: Acute lymphoblastic leukemia; IQR: interquartile range; BMT: Bone marrow transplant; CIMP: CpG island methylator phenotype; WBC: white blood cell.

### Ingenuity pathway analysis

**Fig 2:**
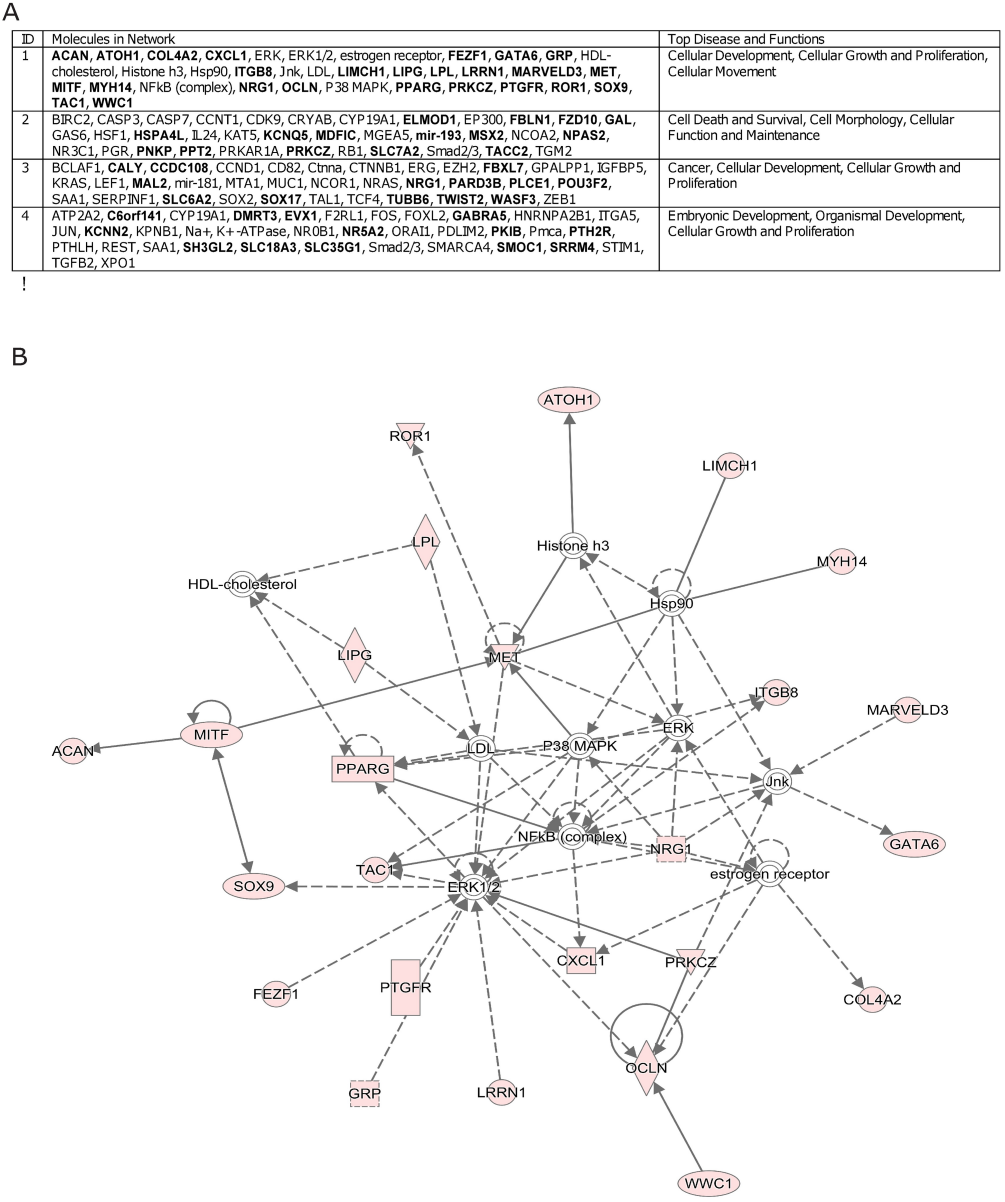
Ingenuity pathway analysis of genes with differentially methylated promoter sites. **(A)** Genes with differentially methylated promoters map to four main networks. Genes shown in bold contain one or more differentially methylated promoters in patients with ALL. **(B)** Graphical depiction of the interactions of genes in network 1.

The 281 promoter sites used for hierarchical clustering aligned to 208 genes (S4 Table). Using the IPA software, we found that these genes were enriched for four main networks with shared functions and diseases in each network: (1) cellular development, cellular growth and proliferation, cellular movement; (2) cell death and survival, cell morphology, cellular function and maintenance; (3) cancer, cellular development, cellular growth and proliferation; and (4) embryonic development, organismal development, cellular growth and proliferation (Fig 2A). In particular, genes with aberrant promotion methylation in network 1 associated with genes with known cancer-related functions: *ERK1/2*, *P38 MAPK*, *JNK*, *ERK*, and *NF-κB* (Fig 2B). These aberrantly methylated genes were also enriched for upstream regulators, such as transcription regulators, cytokines, g-protein coupled receptors, ligand-dependent nuclear receptors, enzymes, and micro-RNAs as listed in S5 Table.

### Maternal *MTR* variants and methylation in children with ALL

Finally, we evaluated the association between maternal *MTR* genotypes and CIMP status in patients with ALL. All SNPs were in Hardy-Weinberg equilibrium and displayed potential functions in terms of overlap with conserved regions, transcription factor binding, regulatory potential or DNaseI hypersensitivity sites (Table 2). Logistic regression was used to calculate odds ratios (OR) and 95% confidence intervals (CI) to evaluate the associations between maternal genotypes and CIMP classification adjusting for ethnicity (non-Hispanic vs. Hispanic). Due to sample size, a dominant model of inheritance was used. Associations between maternal *MTR* variants and CIMP status among cases are presented in Table 3. We found a significant association between maternal *MTR* rs12759827 and CIMP+ phenotype (OR = 4.67, 95% CI: 1.46–16.31) and observed elevated ORs for all other *MTR* SNPs investigated.

**Table 2:** 5-methyltetrahydrofolate-homocysteine methyltransferase (*MTR*) single nucleotide polymorphisms analyzed in the study.

RefSNP	Polymorphism	MAF^a^^,^^b^	Functional consequence^b^	Conserved region^b^^,^^c^	TF binding^b^^,^^c^	Regulatory potential^b^^,^^c^	Dnase I hypersensitivity^b^^,^^c^
rs1050996	2391G>C	0.27	3'UTR	No	No	No	No
rs10733117	2861G>A	0.47	intron variant	No	Yes	No	No
rs10754584	797G>T	0.27	intron variant	No	Yes	Yes	No
rs10802564	1056G>A	0.46	intron variant	No	No	No	No
rs12759827	113A>G	0.09	intron variant	Yes	Yes	Yes	No
rs2282369	170A>G	0.3	intron variant	No	Yes	Yes	Yes
rs2297965	49G>A	0.46	intron variant	Yes	Yes	Yes	No
rs2385511	1847C>A	0.26	intron variant	No	No	No	No
rs3768142	1710G>T	0.36	intron variant	No	Yes	No	No
rs3768150	156T>A	0.27	intron variant	No	Yes	No	No
rs4659745	657A>G	0.34	intron variant	No	Yes	Yes	No

^a^ Minor allele frequency for SNPs (1).

^b^ Per dbSNP (1).

^c^ Potential functions based on publically-available genome-wide datasets from the UCSC Genome Browser (3).

Abbreviations: *MTR*: methionine synthase; MAF: minor allele frequency; TF: transcription factor.

**Table 3:** Association between DNA methylation in children with pediatric all and maternal polymorphisms of *MTR* gene.

RefSNP	OR^a^	95% CI^a^	*P*-value^a^
rs1050996	2.17	0.68–7.54	0.2
rs10733117	2.35	0.70–8.80	0.18
rs10754584	1.73	0.55–5.72	0.36
rs10802564	2.13	0.66–7.40	0.22
rs12759827	4.67	1.46–16.31	0.01
rs2282369	2.02	0.64–6.69	0.24
rs2297965	2.13	0.66–7.40	0.22
rs2385511	1.97	0.63–6.54	0.25
rs3768142	1.22	0.39–3.93	0.73
rs3768150	1.73	0.55–5.72	0.36
rs4659745	2.73	0.82–10.17	0.11

^a^Adjusted for ethnicity.

Abbreviations: MAF: minor allele frequency.

**Fig 3:**
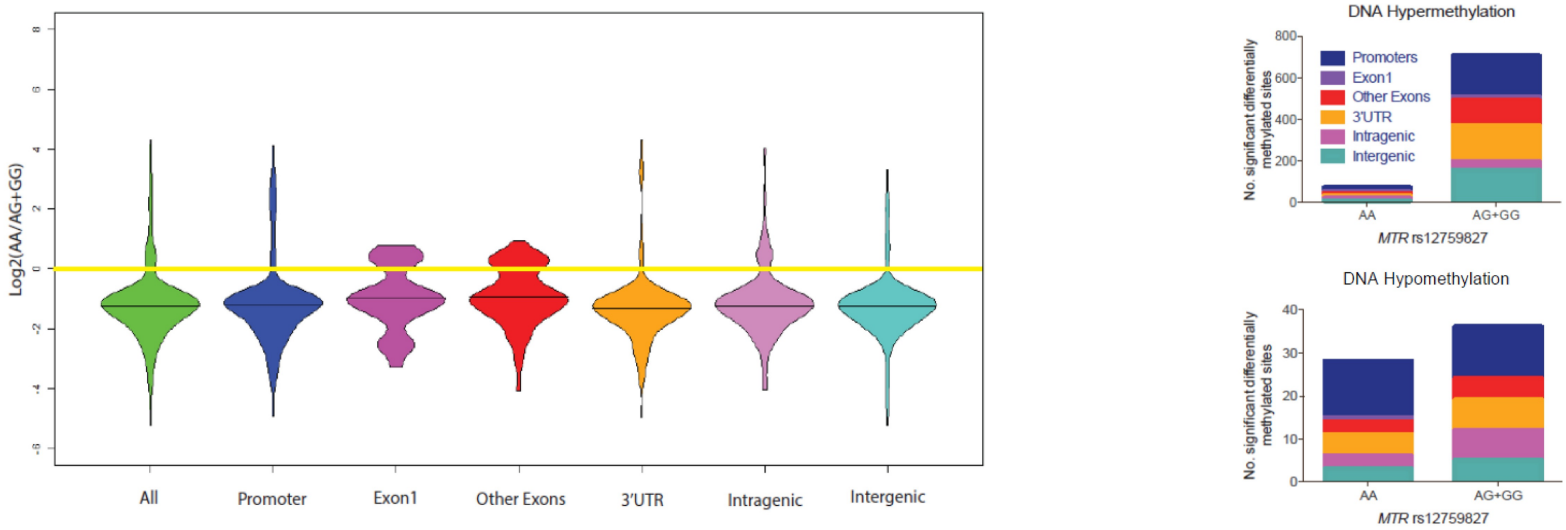
Differential DNA methylation patterns according to maternal *MTR* rs12759827 genotype. **(A)** Log ratio of methylation density comparing children of rs12759827AA (wild-type) mothers to children of AG+GG mothers across all genomic locations. Horizontal black lines represent the log ratio of mean methylation density in cases of AA vs. AG+GG mothers. **(B)** Upper panel: Cases with AA mothers (N = 29) demonstrate DNA hypermethylation at significantly fewer sites than children of AG+GG mothers (N = 22). Lower panel: no association of maternal genotype with number of differentially hypomethylated sites was detected.

We visualized the log ratio of DNA methylation density in children of AA versus AG+GG mothers (Fig 3A). We observed below-zero means for all genomic locations, suggesting that cases with AA mothers displayed lower DNA methylation. Similarly, we demonstrated that cases with AG+GG mothers had a significantly higher number of hypermethylated sites than those with AA mothers (Fig 3B, upper panel), whereas no significant difference was observed in hypomethylated sites (Fig 3B, lower panel).

## Discussion

We previously reported associations of maternal variants in folate metabolic genes with risk of ALL which were independent of offspring genotype. In this study, we sought to determine whether these variants were associated with differences in somatic DNA methylation among children with newly diagnosed ALL. Notably, we identified differential DNA hypermethylation in patients with ALL according to maternal *MTR* genotype. These data suggest that maternal folate metabolism during pregnancy may impact leukemogenesis via DNA methylation. While other studies have indicated that maternal genetic effects (independent of inherited genetic effects) play a role in ALL susceptibility, to our knowledge this is the first study linking maternal genetic effects to somatic epigenetic changes in ALL.

Patients with CIMP+ and CIMP- had similar demographic and clinical profiles; however, the odds of observing maternal rs12759827 was higher among CIMP+ cases. Moreover, patients with ALL and this maternal variant demonstrated DNA hypermethylation across promoters, exons, 3’-UTRs, intragenic and intergenic regions. A similar trend was observed for all other maternal variants, although none of these associations reached statistical significance. While we and others have shown associations of maternal folate metabolic genes with risk of ALL [10, 12], here we extend these observations by providing evidence of association between maternal *MTR* genotype and somatic DNA methylation, a clinically relevant aspect of disease phenotype.

We found that patients with CIMP+ more often harbored the *ETV6-RUNX1* fusion, which is in agreement with previous research [20]. Although this fusion gene can arise *in utero* [8], additional oncogenic events are required for leukemogenesis [21], and DNA hypermethylation events profiled in this study could cooperate in leukemogenesis. This hypothesis is supported by results from Ingenuity Pathway Analysis, which revealed that differentially methylated promoter sites were enriched in genes involved in cell growth, proliferation and survival pathways and which interact with *ERK1/2*, *P38 MAPK*, *JNK*, *ERK*, and *NF-κB*. Furthermore, nearly half (43%) of the cases in this study were classified as CIMP+, suggesting that this phenomenon is not confined to *ETV6-RUNX1* ALL.

This study has certain limitations. Due to sample size, we were not able to evaluate the role of maternal *MTR* genotypes by ALL subtype nor to draw definitive conclusions concerning the association of CIMP status with initial WBC count or cytogenetics. Although we cannot exclude the possibility that these findings occurred due to chance or multiple comparisons, we did note a strong effect of *MTR* rs12759827 on DNA hypermethylation overall and elevated ORs for CIMP+ among children of mothers with two copies of the minor allele at all other SNPs which would not be expected under the null hypothesis. Finally, this study examined only variants in the *MTR* gene, which was previously demonstrated to be associated with ALL, and we restricted our analysis of offspring’s methylation to promoter regions. Further studies will be needed to assess potential effects on DNA methylation of other genes involved in maternal folate transport and metabolism, as well as other genomic regions.

Few studies have evaluated the role of developmental factors on the ALL epigenome. Maternal folate metabolism plays an important role during fetal development; therefore, variation in the maternal genome may potentially impact postnatal leukemia risk, either through establishment of aberrant DNA methylation, or by directly influencing the *in utero* formation of preleukemic transformations such as *ET6V-RUNX1* [22]. Evidence from this work suggests maternal rs12759827 may influence DNA hypermethylation in ALL. Additionally, we show that a high proportion of patients with ALL demonstrate promoter hypermethylation of genes implicated in cell growth and proliferation pathways and that this correlates with specific cytogenetic alterations and other clinical features. Future studies are warranted to assess the effects of the maternal genome on DNA methylation as well as to validate the identified methylation patterns and determine the prognostic and therapeutic significance of DNA methylation in ALL.

## Supporting information

S1 Fig **(A-F)** Observed versus expected % methylation for six genes according to M.SssI treatment. **(G-L)** Correlations between MCA-Seq and pyrosequencing data for six genes.
(PDF)

S1 Table Primer sequences for pyrosequencing. (DOCX)

S2 Table Primer sequences for genotyping maternal single nucleotide polymorphisms in the study. (DOCX)

S3 Table Number of raw and aligned reads for each bone marrow sample. (XLSX)

S4 Table Genes with differentially methylated promoter sites in CIMP+ patients. (XLSX)

S5 Table Upstream regulators enriched for genes aberrantly methylated at promoters in cases. (XLSX)
