# Supplementary figures and images for: Maternal folate genes and aberrant DNA hypermethylation in pediatric acute lymphoblastic leukemia
Source: PLoS One. 2018 May 15;13(5):e0197408. doi: 10.1371/journal.pone.0197408 (PMC5953491; doi:10.1371/journal.pone.0197408)

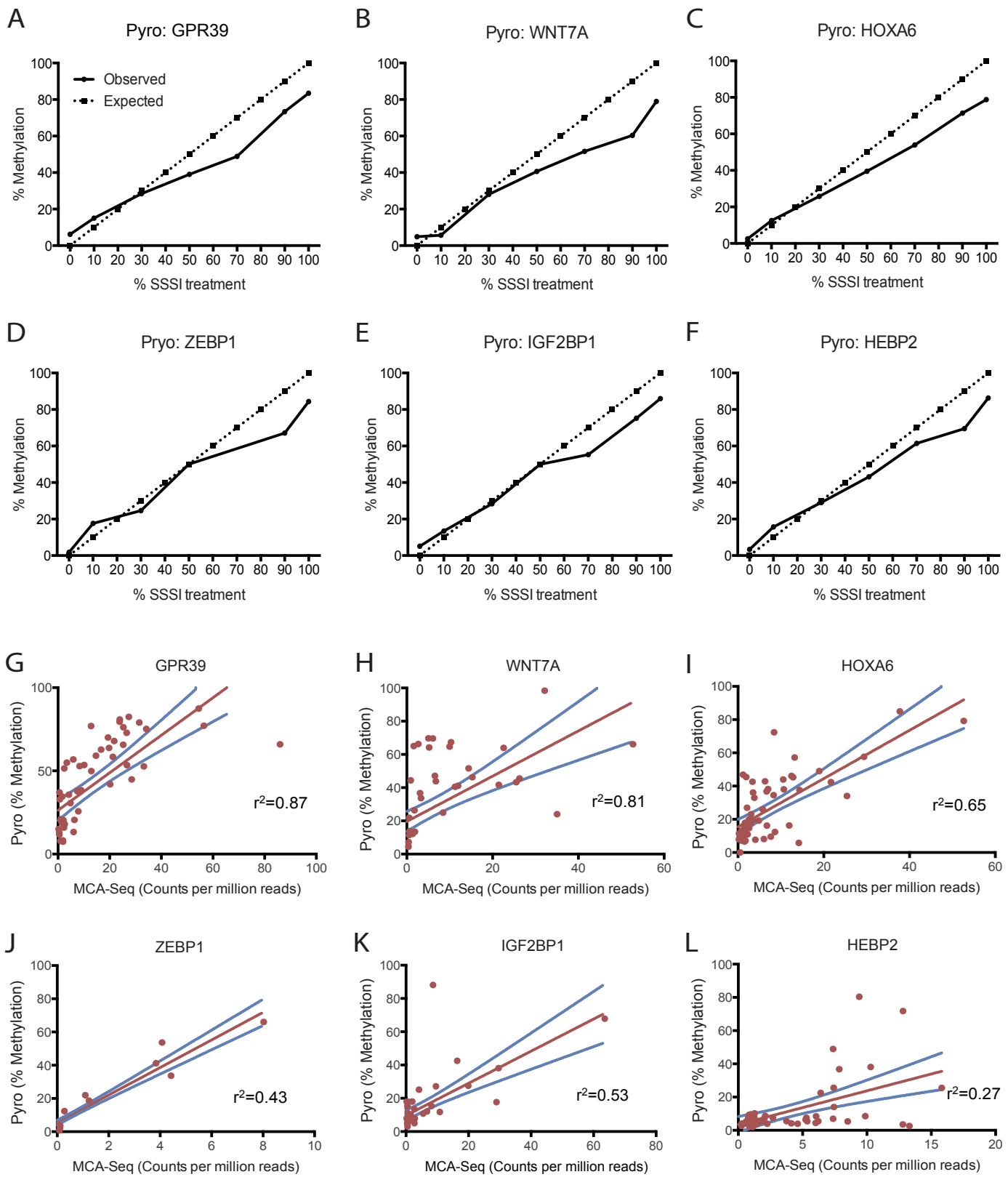

Supplement: S1 Fig — (A-F) Observed versus expected % methylation for six genes according to M.SssI treatment. (G-L) Correlations between MCA-Seq and pyrosequencing data for six genes. (PDF) [file pone.0197408.s001.pdf]
