# Supplementary material for: Maternal folate genes and aberrant DNA hypermethylation in pediatric acute lymphoblastic leukemia
Source: PLoS One. 2018 May 15;13(5):e0197408. doi: 10.1371/journal.pone.0197408 (PMC5953491; doi:10.1371/journal.pone.0197408)
Supplement: S1 Table — (DOCX) [file pone.0197408.s002.docx]

| **S1 Table. Primer Sequences for Pyrosequencing.** | | | |  |
| --- | --- | --- | --- | --- |
| Gene | Forward Primer | Reverse Primer | Sequencing Primer | |
| GPR39 | 5’-AAAGGTAGGGATTTTATAGTGAATTT-3’ | 5’-CAACAAACCCTCCCCTCCTCTATAAAATAT-3 | 5’-ACCAAAAACAACTAAACTCTA-3’ | |
| WNT7A | 5’-AAGGGGTTTTGGGAGTTTAAG-3’ | 5’-ACTAAACAATCCCCAAATAACTAAC-3’ | 5’- GGGTGGTGTTAAGGT -3’ | |
| HOXA6 | 5’-GGGGAAAGTGGGATTTATAAAATAGG-3’ | 5’-ACACAACAAATCACAATCCTACA-3’ | 5’-GTGGGATTTATAAAATAGGAA-3’ | |
| ZEBP1 | 5’-GGGAATTTTTAGGGGAGTTTAGA-3’ | 5’-AATCCCCCCCTACCTACTTCCT-3’ | 5’-TTAGATAGAGATTTTTTTTTTTGGT-3’ | |
| IGF2BP1 | 5’-AATAGGGGAGGGGTGAGTAAT-3’ | 5’-AAATAATCCAACCCAAATCTCACAT-3’ | 5’-GGAGGGGTGAGTAATT-3’ | |
| HEBP2 | 5’-TTAGATAGAGATTTTTTTTTTTGGT-3’ | 5’-CTCCATTCACACTTTCAAACAAACATCTAT-3’ | 5’-GGGAGGGGAGGAATTGG-3’ | |
|  | | | | |
