# Supplementary material for: Maternal folate genes and aberrant DNA hypermethylation in pediatric acute lymphoblastic leukemia
Source: PLoS One. 2018 May 15;13(5):e0197408. doi: 10.1371/journal.pone.0197408 (PMC5953491; doi:10.1371/journal.pone.0197408)
Supplement: S2 Table — (DOCX) [file pone.0197408.s003.docx]

| **S2 Table. Primer Sequences for Genotyping Maternal Single Nucleotide Polymorphisms in the Study.** | | | |  |
| --- | --- | --- | --- | --- |
| RefSNP | Forward Primer | Reverse Primer | Sequencing Primer | |
| rs1050996 | 5’-AAGGTTGGGTCTGACAGTACCAGT-3’ | 5’-CAAGAGTTTGCTGGACATACAGGT-3 | 5’-GTTGGAAGCAAGATCAT-3’ | |
| rs10733117 | 5’-AGCTGGGCATGTAGGTTAGAGTC-3’ | 5’-TTGCAGGTTGGGAAATGTC-3’ | 5’-GGGAAATGTCTTCTAACAG-3’ | |
| rs10754584 | 5’-ACATTGTGCAGGCGAGGA-3’ | 5’-CACTGGAAGTCTCGCAGATCAC-3’ | 5’-AGGCGAGGAAGGTTA-3’ | |
| rs10802564 | 5’-TGGGCTGTGGAGACTACTTAGTT-3’ | 5’-GCACACCTGATAAATTGCATTACT-3’ | 5’-AAGGTGATACTTAGGTTTCC-3’ | |
| rs12759827 | 5’-TTGCACACGTGGTTCCTTTGATA-3’ | 5’-AGGAAGTCAGGGGCCTAGCTACA-3’ | 5’-CTTTGGCTTACGAGTAAC-3’ | |
| rs2282369 | 5’-CTGAGTGGTCCTGCCTCTGA-3’ | 5’-AGCCCAAGCCATCTGTGAG-3’ | 5’-CCATCTGTGAGGACG-3’ | |
| rs2297965 | 5’-AATGCAAGGGAGCGATGAA-3’ | 5’-CCATCTGTGCGTTGTGAAGC-3’ | 5’-CGCCGTTAGCAAGAA-3’ | |
| rs2385511 | 5’-CAGAAACAGCCCATTTGCA-3’ | 5’-GTAGCATTGGTCGCCCCTGT-3’ | 5’-GGTAGACTTTAAAAGTTCAA-3’ | |
| rs3768142 | 5’-CACTGAGGGTTTGCGTATCTT-3’ | 5’-TTATCACACACAAGCCAAAGGAGT-3’ | 5’-GTTTTTGTAAAATGAAAGTA-3’ | |
| rs3768150 | 5’-CGGCTGCTTTCATATTGCTCA-3’ | 5’-TGATGCAATAAGGACCAATCTAGC-3’ | 5’-AACTTTTTAATGGTTTTCAA-3’ | |
| rs4659745 | 5’-AGACAAAGGCCCAGACACCC-3’ | 5’-TTCCGCTGAAAACTGTGAGCTTAG-3’ | 5’-TGACAGAACCAGGACA-3’ | |
